# Diet composition of omnivorous Mesopotamian spiny‐tailed lizards (*Saara loricata*) in arid human‐altered landscapes of Southwest Iran

**DOI:** 10.1002/ece3.9783

**Published:** 2023-01-31

**Authors:** Ali T. Qashqaei, Zeinab Ghaedi, Sean C. P. Coogan

**Affiliations:** ^1^ Borderless Wildlife Conservation Society Tehran Iran; ^2^ Farhangshahr Shiraz Iran; ^3^ Department of Renewable Resources University of Alberta Edmonton Alberta Canada

**Keywords:** arid environments, diet, fecal pellet analysis, omnivore, *Saara loricata*, seed dispersal

## Abstract

The Mesopotamian spiny‐tailed lizard, *Saara loricata*, is one of the largest lizard species in the Middle East. Here, we report on the diet of the lizard and their potential role in seed dispersal in Southwestern Iran. We analyzed lizard fecal pellet groups (*n* = 124) for their food item composition and seed content. We calculated the relative frequency of occurrence (FO%), relative volume (V%), and importance value (IV%) for each food item. Moreover, the number of seeds of each plant food item was counted. Our findings reveal the first solid evidence of omnivorous behavior in the lizard. In total, 16 plant food items and 14 animal food items were identified. Herbaceous plants (IV = 110.2%) and invertebrates (4.8%) were the most important food groups. The plant food items with the highest FO% were Poaceae (56.4%), *Centaurea* sp. (43.5%), and *Medicago polymorpha* (27.4%); and the V% for these items were 53.6%, 30.9%, and 13.1%, respectively. Most of the seeds that were consumed by lizards were from Poaceae (547 seeds; 47.81%) and Fabaceae (285 seeds; 24.91%). We also found that each individual lizard could play an equal role in the seed dispersal of all plant families identified. Previous studies show that plant species density and richness are important features for the burrow site selection of Mesopotamian spiny‐tailed lizard. This study highlights the potential role of lizards in influencing the vegetation communities around their burrows through seed dispersal.

## Graphical abstract

The Mesopotamian spiny‐tailed lizard, *Saara loricata* (Blanford, 1874), is a desert dweller, one of the largest lizard species in the Middle East and occurs in Southwestern Iran and Southeastern Iraq.

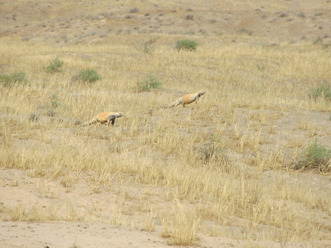

## 1 INTRODUCTION

Knowledge of species' feeding behavior and their role in seed dispersal are important in understanding ecosystem functioning and the regeneration of plant communities (Pianka, 1986; Wenny & Levey, 1998). Many studies have documented the role of mammals and birds in seed dispersal of plant communities (Tsunamoto et al., 2020). For instance, seed dispersal by birds and mammals has been documented in central Japan, Southeastern Iran, Mediterranean habitats, and North America (Fahimi et al., 2018; Herrera, 1989; Naoe et al., 2018; Sayedi et al., 2022; Spennemann, 2020; Traveset & Willson, 1997). However, studies on the role of lizards in dispersing plant seeds in arid environments have rarely been undertaken (Iverson, 1985; Valido & Olesen, 2019).

Lizards are important seed dispersers (Valido & Olesen, 2019). In fact, lizards have been shown to be even more effective than birds for dispersing seeds in some plant communities, especially on islands (e.g., Galápagos and Canary Islands) where other seed dispersers have disappeared or experienced significant population declines (González‐Castro et al., 2015; Heleno et al., 2013). Lizards are the earliest mutualistic vertebrates with plants since the Paolozoic Era (541–251 million years ago) and play an important role in seed dispersal (Tiffney, 2004; Willson et al., 1996). Lizards are often opportunistic animals in their foraging behavior (Griffiths & Christian, 1996) and the larger species and generalist lizards eat more fruits and more seeds and have greater potential for seed dispersal (Miranda, 2017; Valencia‐Aguilar et al., 2013; Valido & Olesen, 2019).

**FIGURE 1:**
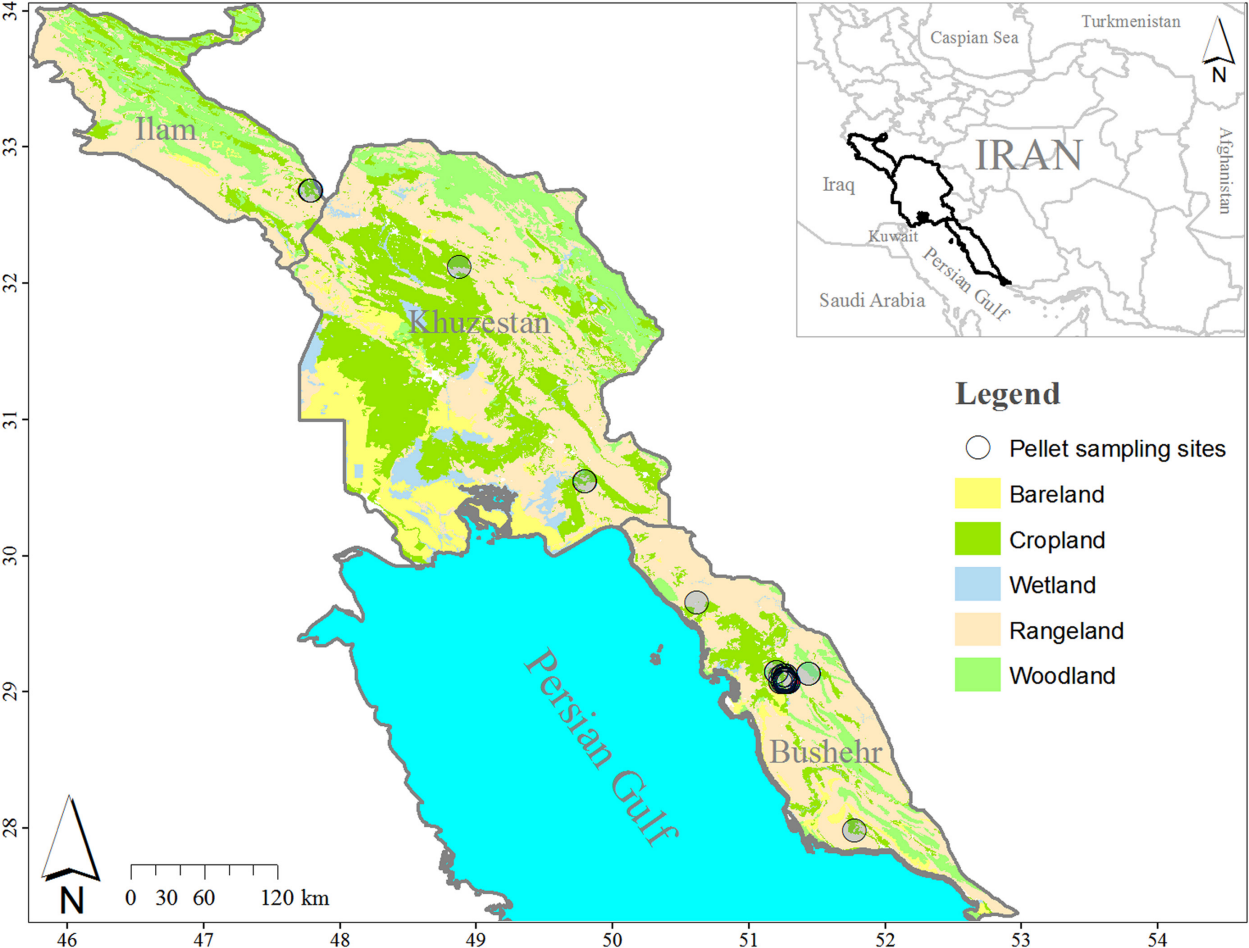
Sampling site locations of fecal pellet groups of Mesopotamian spiny‐tailed lizard, *Saara loricata*, in Iran.

The Mesopotamian spiny‐tailed lizard (hereafter Mesopotamian lizard) *Saara loricata* (Blanford, 1874) is a desert dweller, one of the largest lizard species in the Middle East, and occurs in Southwestern Iran and Southeastern Iraq (Figure 1, Anderson, 1999; Ghaedi et al., 2021; Kafash et al., 2016; Papenfuss et al., 2009; Safaei‐Mahroo et al., 2015). Anderson (1999) reported that Mesopotamian lizards tend to be completely herbivorous. However, Nazari‐Serenjeh and Torki (2017) have mentioned predating some animal items by Mesopotamian lizards. The Mesopotamian lizard typically lives in burrows (typically one lizard per burrow) in arid lands with sparse vegetation, annual and perennial plant species, and its habitat is similar to that of the Egyptian spiny‐tailed lizard (*Uromastyx aegyptia*) in Western Asia (Ghaedi et al., 2021; Nazari‐Serenjeh & Torki, 2017; Papenfuss et al., 2009; Wilms et al., 2009; Zohary, 1973). The Mesopotamian lizard in Iran predominantly occurs in human‐altered landscapes such as farmlands (Ghaedi et al., 2021; Nazari‐Serenjeh & Torki, 2017).

In this study, we examined the diet of the Mesopotamian lizard in Southwestern Iran to determine whether they have an herbivorous or omnivorous diet. Moreover, it is important to study the feeding behavior of Mesopotamian lizards in Iran because their habitat has a high degree of human disturbance which may impact their diet. For example, about 20%–30% of areas in Western‐Southwestern Iran are suitable habitats for Mesopotamian lizards (Kafash et al., 2016; Rastegar‐Pouyani & Fathinia, 2015); however, these areas are mostly degraded and fragmented by human activities (e.g., overgrazing, cultivation, and oil mulching) (Elhaeesahar & Masoudi, 2018; Ghaedi et al., 2021; Mohammad Rahimi et al., 2019). Furthermore, the Mesopotamian lizards may provide important ecological benefits in the region through seed dispersal; however, we need a better understanding of whether the lizards may act as seed dispersers of native vegetation and which species of plants are likely to be dispersed. Thus, our study of the Mesopotamian lizard's diet, and knowledge regarding their potential role in seed dispersal, is important for the rehabilitation of their habitat. In this study, our aims were as follows: (a) to study the diet composition of the Mesopotamian lizard in Iran using fecal pellet analysis to determine whether they are herbivorous or omnivorous; (b) to measure whether and to what extent Mesopotamian lizards have the potential to disperse seeds of various plant species consumed; and (c) to determine whether the number of seeds ingested varies among individuals.

## 2 MATERIAL AND METHODS

### 2.1 Study area

We conducted our study across three provinces in Southwestern Iran, including Bushehr, Khuzestan, and Ilam, which collectively cover an area of 107,553 km^2^ (Kafash et al., 2016, Figure 1). The average annual precipitation ranges between 200 and 266 mm and the maximum temperature reaches up to 59°C (Stone, 2016; Zhao et al., 2021). The vegetation of the area is characterized by open scattered scrublands dominated by wild jujube (*Ziziphus nummularia*), Christ's thorn jujube (*Ziziphus spina‐christi*), salam (*Acacia ehrenbergiana*), umbrella thorn (*A. tortilis*), orfot (*A. oerfota*), gum arabic tree (*A. nilotica*), and ghaf (*Prosopis cineraria*) (Dinarvand, 2021).

### 2.2 Study design

We located our sampling areas based on the occurrence of the Mesopotamian lizard, these areas lie mainly around croplands (Figure 1, Nazari‐Serenjeh & Torki, 2017). In each sampling site, we searched for burrows of the Mesopotamian lizards. To ensure independent sampling of individual lizards, we retained approximately ≥100 meters distance between lizard burrows. This distance was taken from the average home range (1 to 5 hectares) of similar species such as the North‐African spiny‐tailed lizards, *Uromastyx acanthinura* (Chiraz, 2021). We selected 24 sites (24 burrow sites of 24 individuals).

### 2.3 Field and laboratory work

From May to July 2016, we collected fresh (approximately 1‐week‐old) fecal pellet groups (several pellets together) around each lizard burrow (a distance of 10 m from each burrow) and stored them in plastic bags. In the laboratory, we counted the number of pellets in each pellet group for each individual lizard and measured the diameter and the length of each pellet separately by caliper. We also calculated the dry weight of each pellet using a digital scale. Finally, we counted the number of pellets in each pellet group. We soaked the pellet groups in water for approximately 20–40 min. We then removed the pellets and placed them in water inside a petri dish and separated food items using tweezers under a Nikon stereo microscope. We divided food items into two groups: animal and plant food items. We identified the consumed food items to the lowest taxa using identification keys wherever possible. Within the animal food category, we further categorized food items as vertebrates and invertebrates. Likewise, items in the plant food category were further categorized as being cultivated trees, wild fruits, herbs, shrubs, and crops. We counted the seeds of the grasses, herbs, shrubs, and fruits in each pellet group.

### 2.4 Statistical analysis

We calculated the relative frequency of occurrence (FO%) of the food items in each pellet group (Ghadirian et al., 2017; Soofi et al., 2017):

$$\begin{array}{c} {\mathrm{FO}}_{\text{item}}\%=\frac{\text{Number of pellets in which food item}\;\left(i\right)\;\text{occurred}}{\text{Total number of pellets}}\times 100. \end{array}$$

Next, we measured the volumetric percentage of the identified food items as two‐thirds (66.6%), half (50%), one‐third (33.3%), one‐fourth (25%), and trace (≤1%). The measured volume of each food item in each pellet group was placed into six groups for estimating mean volume: trace (≤1%), 1%–25%, 25%–50%, 50%–75%, 75%–100%, and 100%. The mean point of each volumetric group was used to calculate the overall percentage of the identified food items in the pellets (Ghadirian et al., 2017; Soofi et al., 2017). Furthermore, we calculated the relative volume (V%) of the identified food items in the pellets. In addition, we calculated the importance value (IV%; an estimate of the importance of consumed foods) of the food items/groups estimated (Ghadirian et al., 2017; Soofi et al., 2017):

$$V_{\text{item}}\%=\frac{\sum \text{percent of volume of item}\;\left(i\right)\;\text{in pellet group}\;\left(j\right)}{\text{number of pellet group in which food item}\;\left(i\right)\;\text{occurred}}$$

$${\mathrm{IV}}_{\text{item}}\%=\frac{\mathrm{FO}\%\text{of item}\;\left(i\right)\times V\%\text{of item}\;\left(i\right)}{100}$$

where, item *i* is the specific consumed food item and pellet group *j* is the specific pellet group. To compare the difference in the mean ranks of the food groups consumed by lizards, we ran a Dunn's test, which applies a Kruskal–Wallis rank‐sum test for stochastic dominance among multiple groups (Dinno, 2017). The mean ranks between different lizard individuals and the seed dispersal of different plant families were compared using Tukey honest test (Dytham, 2011).

## 3 RESULTS

Overall, our sampling led to the collection of 440 pellets in 124 pellet groups. The mean number of fecal pellet groups per lizard was 5.16 ± 6.48 SD (range 1–31). The mean number of fecal pellets in each pellet group was 3.7 ± 2.4 SD (range 1–13) and the mean weight (g) of the pellet groups was 1.1 ± 1.0 SD (range 0.11–5.87). Furthermore, the mean number of food items in each pellet group was 2.4 ± 1.3 SD (range 1–7), and the food items consumed by the individual lizards ranged between 1 and 11 items (Table S1).

We identified 16 plant food items and 14 animal food items in the pellet groups of the Mesopotamian lizards. Of the total analyzed pellet groups, 69 (55.65%) exclusively contained animal food items, and only a single (0.8%) pellet group contained entirely plant food items. However, 43.55% (*n* = 54) of the pellet groups included both plant and animal food items. The FO% of the plant food items (93.7%–100%) was much greater than animal food items (43.7%–44.7%) across pellet groups (Tables 1 and 2).

**TABLE 1:** Frequency of occurrence of plant and animal items in lizard fecal pellet groups in the three different provinces in the study area.

Food group	Bushehr province (*n* = 76)				Khuzestan province (*n* = 16)				Ilam province (*n* = 32)			
	No.	FO%	V%	IV%	No.	FO%	V%	IV%	No.	FO%	V%	IV%
Animal food items	34	44.7	3.3	1.5	7	43.7	12.4	5.4	14	43.7	2.4	1.0
Plant food items	76	100	96.7	96.7	15	93.7	87.6	82.1	32	100	97.6	97.6

*Note*: Here, FO%, V%, and IV% are the frequency of occurrence of items, volume percent of items, and importance value of items, respectively.

**TABLE 2:** Food items identified (animal and plant food items) in fecal pellet groups (*n* = 124) collected from Southwestern Iran, May–July 2016.

Identified food item	Food group	FO%	V%	IV%
*Animal food items*	*Animal food group*	*44.3*	*4.2*	*1.9*
Snail, Gastropoda	Invertebrates	0.8	0.01	1>
Unidentified insect	Invertebrates	2.4	0.02	1>
Unidentified beetle	Invertebrates	37.9	2.01	1>
Ant, Formicidae	Invertebrates	3.2	0.6	1>
Scorpion, Scorpiones	Invertebrates	0.8	0.04	1>
True weevils, Curculionidae	Invertebrates	0.8	0.01	1>
Cockroaches, Dictyoptera	Invertebrates	0.8	0.2	1>
Ground beetle, Carabidae	Invertebrates	3.2	0.4	1>
Jewel beetle, Buprestidae	Invertebrates	1.6	0.32	1>
Adesmia beetle, *Adesmia* sp.	Invertebrates	0.8	0.4	1>
Fly, Diptera	Invertebrates	0.8	0.01	1>
Wasp, Hymenoptera	Invertebrates	0.8	0.04	1>
Moth, Lepidoptera	Invertebrates	0.8	0.04	1>
Small mammals	Vertebrates	4.8	0.16	1>
*Plant food items*	*Plant food group*	*99.2*	*95.8*	*95.0*
Date palm fruit, *Phoenix dactylifera*	Cultivated trees	1.6	1	1>
Fig fruits, *Ficus carica*	Cultivated trees	0.8	0.24	1>
Christ's thorn jujube fruits*, Ziziphus spina‐christi*	Wild trees	5.6	3.35	1>
Rhamnaceae fruits	Wild trees	2.4	1.4	1>
Burr medic, *Medicago polymorpha*	Herbaceous plants	27.4	13.1	3.6
Asteraceae	Herbaceous plants	12.1	7.5	1>
Centaury, *Centaurea* sp.	Herbaceous plants	43.5	30.9	13.1
Fabaceae	Herbaceous plants	12.1	54.2	6.5
Poaceae	Herbaceous plants	56.4	53.6	30.2
Lamiaceae	Herbaceous plants	0.8	0.01	1>
Dandelion, *Taraxacum* sp.	Herbaceous plants	2.4	0.92	1>
Brome grass, *Bromus* sp.	Herbaceous plants	8.1	3.1	1>
Mesquite, *Prosopis juliflora*	Shrub/invasive species	0.8	0.01	1>
Muskmelons, *Cucumis melo*	Crop	0.8	0.03	1>
Common wheat, *Triticum aestivum*	Crop	1.6	0.9	1>
Barley, *Hordeum vulgare*	Crop	0.8	0.8	1>

*Note*: Here, FO%, V%, and IV% are the frequency of occurrence of items, volume percent of items, and importance value of items, respectively.

**FIGURE 2:**
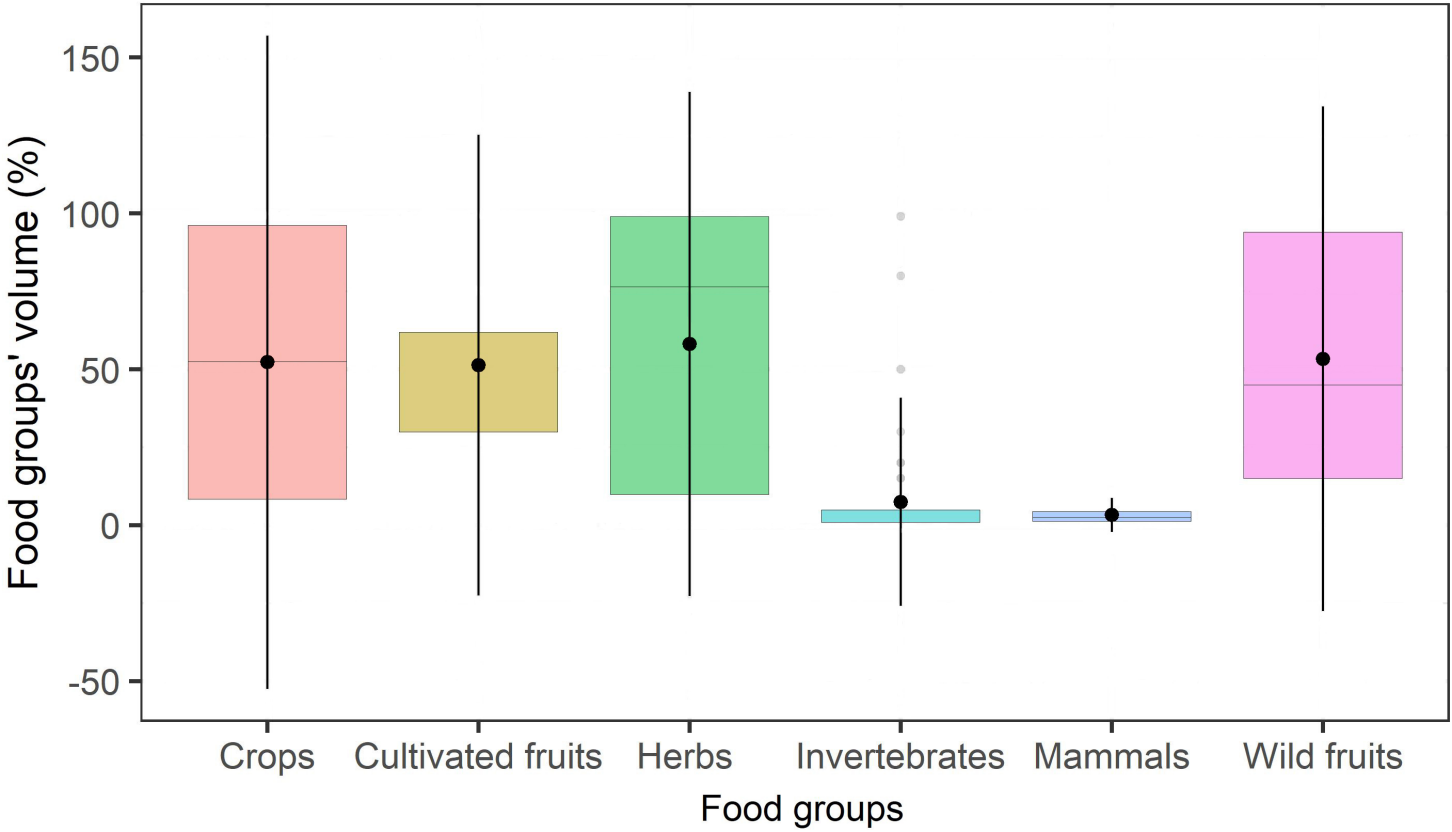
The volume (%) of different food groups identified in the fecal pellet groups (*n* = 124) of the Mesopotamian spiny‐tailed lizard, *Saara loricata*.

We found that herbaceous plants (FO% = 97.6) and invertebrates (FO% = 42.7) had the highest FO% of food groups in the lizards' fecal pellets. Also, multiple comparisons of all food groups showed that the contribution of the invertebrates was significantly (*Z* = 9.61, *p* = .01) different from those of herbaceous plants. Likewise, the difference between consumed small mammals and herbaceous plants was also significant (*Z* = 3.38, *p* = .01). Wild fruits were consumed significantly differently (*Z* = −3.64, *p* = .01) compared to invertebrates (Figure 2). We did not find significant differences among other food groups and no significant difference was found in the mean ranks of the food groups across study sites.

**FIGURE 3:**
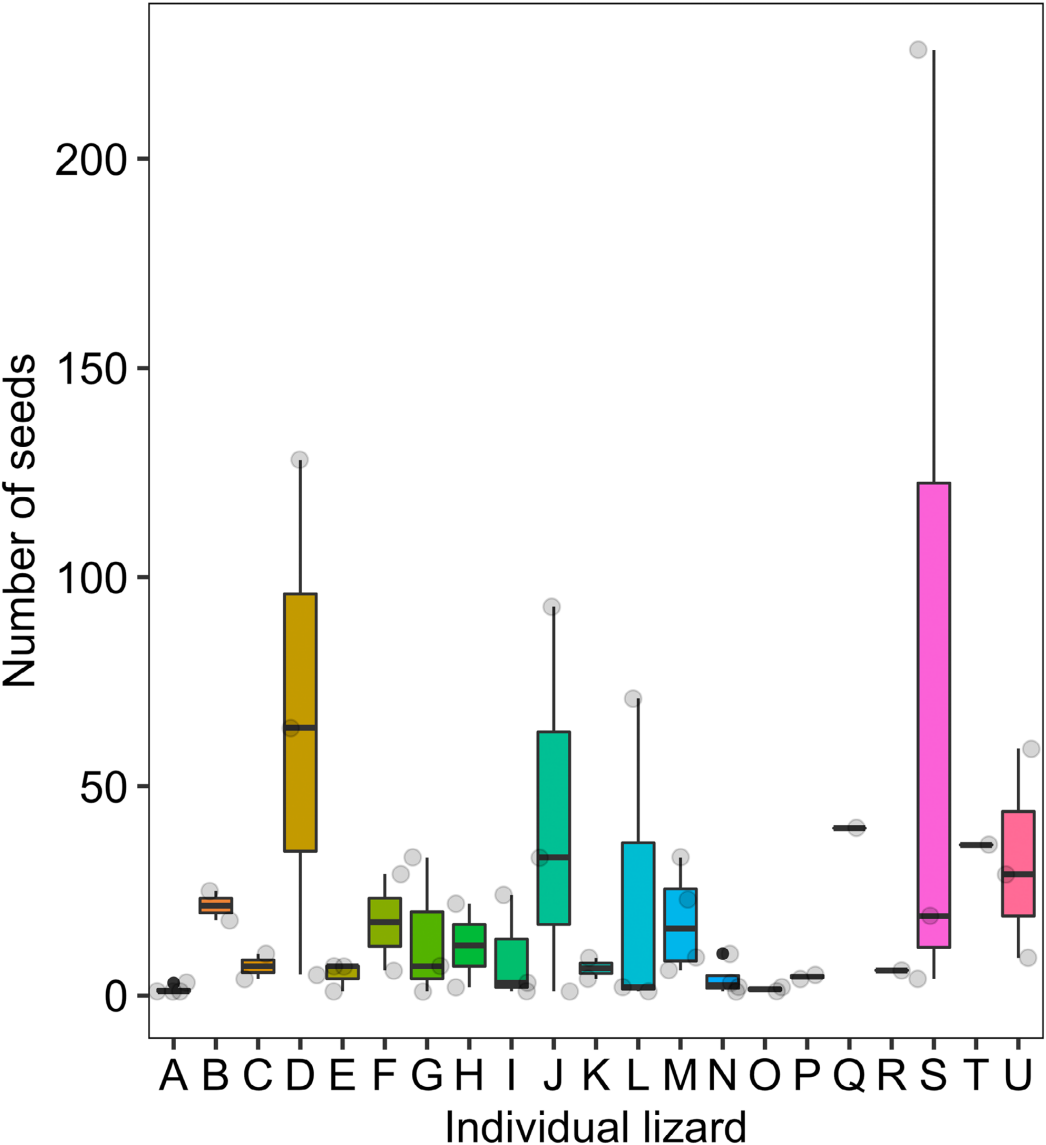
Number of seeds of different plant items identified in the fecal pellet groups of each individual Mesopotamian spiny‐tailed lizard, *Saara loricata*. Each capital letter (A through U) represents an independent individual lizard.

**FIGURE 4:**
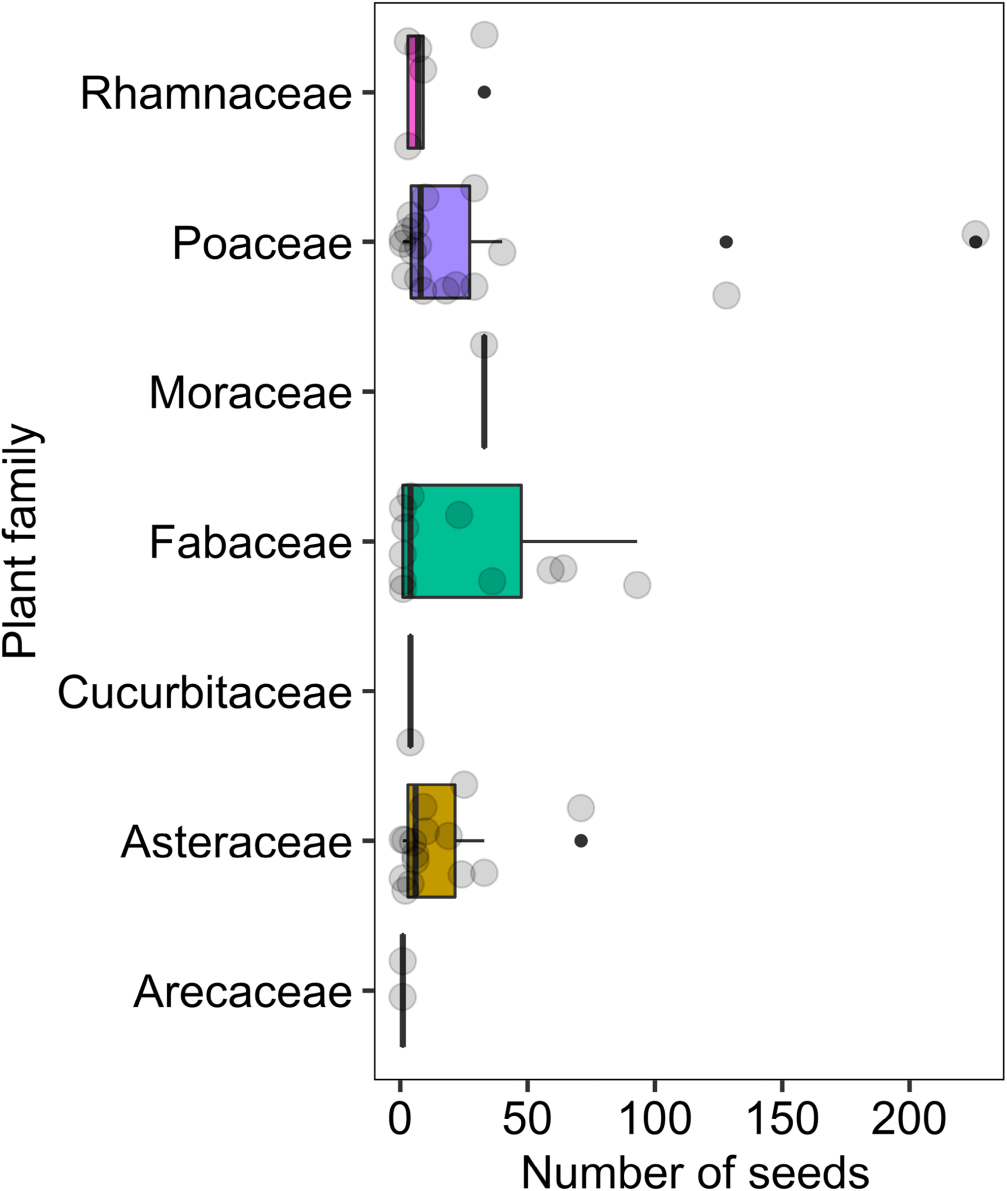
Number of seeds from different plant families (*n* = 7) identified in the fecal pellet groups (*n* = 124) of the Mesopotamian spiny‐tailed lizard, *Saara loricata* in Southwestern Iran.

The majority of seeds in the pellet samples belonged to Poaceae (*n* = 315), *Bromus* sp. (*n* = 226), *Centaurea* sp. (*n* = 158), *Medicago polymorpha* (*n* = 151), and Fabaceae (*n* = 133). The least number of seeds in the samples were related to mesquite (*n* = 1), barley *Hordeum vulgare* (*n* = 2), and the cultivated date palm fruits (*n* = 2). Our results also show that the Mesopotamian lizards ingested 1144 seeds including from wild plants (trees and herbaceous plants, *n* = 1098), cultivated trees and crops (*n* = 45), and invasive plant species (mesquite, *n* = 1, Table 3). The average number of seeds ingested by each individual was 47.6 ± 12.9 SE (range 0–249); only four pellet groups, belonging to three individuals, did not contain seeds. The comparison of ingested seed rates among individual lizards showed that only two individuals (“S” and “D”) were significantly different (*p* ≤ .01). In fact, the number and variety of seeds ingested by individual S and individual D were more than the other individuals (Table S1, Figure 3). The seeds of Arecaceae were consumed significantly less than other plant families (Figure 4, Tables S2 and S3).

**TABLE 3:** Number and frequency (FO%) of seed per plant food item in fecal pellet groups (*n* = 124) collected from Southwestern Iran, May–July 2016.

Family	Plant food item	Bushehr	Khuzestan	Ilam	Total	
		No.	No.	No.	No.	FO%
Poaceae	Barley, *Hordeum vulgare*			2	2	0.17
	Common wheat, *Triticum aestivum*			4	4	0.35
	Brome grass, *Bromus* sp.		226		226	19.75
	Unidentified grass	292		23	315	27.53
Fabaceae	Burr medic, *Medicago polymorpha*	53	40	58	151	13.2
	Mesquite, *Prosopis juliflora*	1			1	0.1
	Unidentified wild legume	132		1	133	11.62
Asteraceae	Centaury, *Centaurea* sp.	152	2	4	158	13.81
	Unidentified daisy	38	17	5	60	5.24
Cucurbitaceae	Muskmelons, *Cucumis melo*	4			4	0.35
Rhamnaceae	Christ's thorn jujube, *Ziziphus spina‐christi*	48			48	4.2
	Unidentified buckthorn fruits	7			7	0.61
Moraceae	Fig fruits, *Ficus carica*	33			33	2.9
Arecaceae	Date palm fruit, *Phoenix dactylifera*	2			2	0.17
Total		762	285	97	1144	100

## 4 DISCUSSION

In this study, we have demonstrated that *S. loricata* has an omnivorous diet in southwestern Iran. In the past, however, the lizard was thought to be herbivorous. The percent of plant material in the pellets of the Mesopotamian spiny‐tailed lizards was higher than animal‐based food items, but pellet groups that contained exclusively plant material were lower than that reported for the Egyptian spiny‐tailed lizard *U. aegyptia* (Castilla et al., 2011; Cunningham, 2000, 2001).

Only, two seeds of date palm (Arecaceae; 0.17% of total seeds) were found in two pellet groups, which is similar to the findings of Cunningham (2000) regarding the Egyptian spiny‐tailed lizards in the United Arab Emirates. Arecaceae (date palm), Cucurbitaceae (Muskmelons, *Cucumis melo* = 0.35% of total seeds), Moraceae (common fig = 2.9% of total seeds), and Rhamnaceae (4.8%) had the least seeds in the Mesopotamian lizard pellets. Cunningham (2009) recorded browsing by Egyptian spiny‐tailed lizard on *Acacia tortilis* shrub in Saudi Arabia, which is also similar to our findings relating to the leaves and legumes of *Prosopis juliflora* (Fabaceae) in *S. loricata* pellets. Most of the seeds that were ingested by the lizards belonged to Poaceae (547 seeds; 47.81%), Fabaceae (285 seeds; 24.91%), and Asteraceae (218 seeds; 19.06%) families. Furthermore, browsing on *Prosopis juliflora* and feeding on wheat and barley (Poaceae) around farmlands has been reported in Bushehr province (Nazari‐Serenjeh & Torki, 2017).

Different insects such as beetles and ants recovered from the Mesopotamian lizard pellets are consistent with reported predation on other insects such as locusts in Bushehr province, Southwestern Iran (Nazari‐Serenjeh & Torki, 2017). According to Subramanean and Vikram Reddy (2012) one of the most important ecological roles of the lizards is the control of pest (e.g., beetles and locusts) around farmlands. Due to the nocturnal life of small mammals and scorpions, and the mid‐day activity of *S. loricata*, it is unlikely that such animals will be predated at mid‐day. However, small mammals and scorpions could enter lizard burrows at night and could be consumed opportunistically. According to Fathinia and Rastegar‐Pouyani (2011) and Ghaedi et al. (2021), the Baluch Rock Geckos, *Bunopus tuberculatus*, Olivier's Agama, *Trapelus ruderatus*, and snakes sometimes live with *S. loricata* in the same burrow.

Kafash et al. (2014) identified that the richness and the density of plant species are very important components for burrow site selection of *S. loricata*. However, seed dispersal by the lizards likely influences the vegetation communities around their burrows. Much of the lizard's habitat occurs in areas with frequent dust storms caused by overgrazing, habitat destruction, and drought (Dinarvand & Jamzad, 2020; Heidarian et al., 2017). Due to rampant poaching of the native large mammalian herbivores such as Persian gazelle (*Gazella subgutturosa*), Indian gazelle (*Gazella bennettii*), and Persian onager (*Equus hemionus*) have vanished in many parts of Western and Southwestern Iran (Hatt, 1959; Soofi et al., 2022). Based on the seeds excreted by the Mesopotamian lizard, this species has the potential to be a seed disperser, which may be especially important in human‐altered habitats (e.g., overgrazed pastures) in the absence of mammalian herbivores.

In light of our study findings that the Mesopotamian lizard has the potential to be an important seed disperser in Southwestern Iran, we suggest that future research should be undertaken to evaluate its actual role in seed dispersal. For example, further research on the survival, germination, dispersal distance of excreted seeds, and seedling establishment should be conducted for a better understanding of this species’ role in seed dispersal in arid environments. Furthermore, it would be interesting to examine seed dispersal by other species to determine to what extent the lizards provide this ecosystem service relative to other species.

## AUTHOR CONTRIBUTIONS

**Ali T. Qashqaei:** Conceptualization (lead); data curation (lead); formal analysis (lead); investigation (lead); methodology (lead); project administration (lead); resources (lead); software (equal); supervision (lead); validation (lead); visualization (lead); writing – original draft (lead); writing – review and editing (lead). **Zeinab Ghaedi:** Conceptualization (supporting); data curation (supporting); formal analysis (supporting); investigation (supporting); methodology (supporting); project administration (supporting); resources (supporting); software (equal); supervision (supporting); validation (supporting); visualization (supporting); writing – original draft (supporting); writing – review and editing (supporting). **Sean C. P. Coogan:** Conceptualization (supporting); data curation (supporting); formal analysis (supporting); investigation (supporting); methodology (supporting); project administration (supporting); supervision (supporting); validation (supporting); visualization (supporting); writing – original draft (supporting); writing – review and editing (equal).

## Supporting information


Appendix S1

## Data Availability

The data that supports the findings of this study are available in the supplementary material of this article.
