# Supplementary material for: Diet composition of omnivorous Mesopotamian spiny‐tailed lizards (Saara loricata) in arid human‐altered landscapes of Southwest Iran
Source: Ecol Evol. 2023 Jan 31;13(2):e9783. doi: 10.1002/ece3.9783 (PMC9889844; doi:10.1002/ece3.9783)
Supplement: Supplementary file 1 — Appendix S1 [file ECE3-13-e9783-s001.docx]

Supplementary Material

**Diet composition of omnivorous Mesopotamian spiny-tailed lizards (*Saara loricata*) in arid human-altered landscapes of Southwest Iran**

Ali T. Qashqaei, Zeinab Ghaedi, Sean C. P. Coogan

Ecology and Evolution

Table S1. Number of collected fecal pellet group, identified food items, counted seeds for each individual lizard during May to July 2016.

| Individual lizard | Code | No. pellet group | No. item in all pellet group | No. seed in all pellet group |
| --- | --- | --- | --- | --- |
| Lizard Number1 | A | 4 | 9 | 6 |
| Lizard Number2 | B | 4 | 4 | 43 |
| Lizard Number3 | C | 1 | 2 | 14 |
| Lizard Number4 | D | 10 | 8 | 197 |
| Lizard Number5 |  | 2 | 3 | 0 |
| Lizard Number6 | E | 4 | 7 | 15 |
| Lizard Number7 | F | 4 | 4 | 35 |
| Lizard Number8 | G | 3 | 10 | 41 |
| Lizard Number9 | H | 3 | 2 | 24 |
| Lizard Number10 | I | 7 | 4 | 28 |
| Lizard Number11 | J | 6 | 6 | 127 |
| Lizard Number12 | K | 1 | 3 | 13 |
| Lizard Number13 | L | 4 | 5 | 74 |
| Lizard Number14 | M | 9 | 6 | 71 |
| Lizard Number15 | N | 3 | 5 | 16 |
| Lizard Number16 | O | 2 | 4 | 3 |
| Lizard Number17 | P | 1 | 2 | 9 |
| Lizard Number18 | Q | 5 | 1 | 40 |
| Lizard Number19 | R | 1 | 2 | 6 |
| Lizard Number20 |  | 1 | 2 | 0 |
| Lizard Number21 | S | 15 | 11 | 249 |
| Lizard Number22 | T | 1 | 3 | 36 |
| Lizard Number23 |  | 1 | 2 | 0 |
| Lizard Number24 | U | 31 | 10 | 97 |

Table S2. Number of dispersed seed for each plant family by individual lizard during May to July 2016.

| Individual of Lizard | family | Seed Number | Location | County | Province | Lon | Lat |
| --- | --- | --- | --- | --- | --- | --- | --- |
| 1 | Arecaceae | 1 | Gandomriz | Borazjan | Bushehr | 51.1959893 | 29.14859746 |
| 1 | Asteraceae | 1 | Gandomriz | Borazjan | Bushehr | 51.1959893 | 29.14859746 |
| 1 | Cucurbitaceae | 0 | Gandomriz | Borazjan | Bushehr | 51.1959893 | 29.14859746 |
| 1 | Fabaceae | 1 | Gandomriz | Borazjan | Bushehr | 51.1959893 | 29.14859746 |
| 1 | Lamiaceae | 0 | Gandomriz | Borazjan | Bushehr | 51.1959893 | 29.14859746 |
| 1 | Poaceae | 0 | Gandomriz | Borazjan | Bushehr | 51.1959893 | 29.14859746 |
| 1 | Rhamnaceae | 3 | Gandomriz | Borazjan | Bushehr | 51.1959893 | 29.14859746 |
| 1 | Moraceae | 0 | Gandomriz | Borazjan | Bushehr | 51.1959893 | 29.14859746 |
| 2 | Arecaceae | 0 | Gas Station, Dilashekan Valley | Borazjan | Bushehr | 51.259657 | 29.10099571 |
| 2 | Asteraceae | 25 | Gas Station, Dilashekan Valley | Borazjan | Bushehr | 51.259657 | 29.10099571 |
| 2 | Cucurbitaceae | 0 | Gas Station, Dilashekan Valley | Borazjan | Bushehr | 51.259657 | 29.10099571 |
| 2 | Fabaceae | 0 | Gas Station, Dilashekan Valley | Borazjan | Bushehr | 51.259657 | 29.10099571 |
| 2 | Lamiaceae | 0 | Gas Station, Dilashekan Valley | Borazjan | Bushehr | 51.259657 | 29.10099571 |
| 2 | Poaceae | 18 | Gas Station, Dilashekan Valley | Borazjan | Bushehr | 51.259657 | 29.10099571 |
| 2 | Rhamnaceae | 0 | Gas Station, Dilashekan Valley | Borazjan | Bushehr | 51.259657 | 29.10099571 |
| 2 | Moraceae | 0 | Gas Station, Dilashekan Valley | Borazjan | Bushehr | 51.259657 | 29.10099571 |
| 3 | Arecaceae | 0 | Samal Abad | Borazjan | Bushehr | 51.2605813 | 29.09663446 |
| 3 | Asteraceae | 10 | Samal Abad | Borazjan | Bushehr | 51.2605813 | 29.09663446 |
| 3 | Cucurbitaceae | 0 | Samal Abad | Borazjan | Bushehr | 51.2605813 | 29.09663446 |
| 3 | Fabaceae | 0 | Samal Abad | Borazjan | Bushehr | 51.2605813 | 29.09663446 |
| 3 | Lamiaceae | 0 | Samal Abad | Borazjan | Bushehr | 51.2605813 | 29.09663446 |
| 3 | Poaceae | 4 | Samal Abad | Borazjan | Bushehr | 51.2605813 | 29.09663446 |
| 3 | Rhamnaceae | 0 | Samal Abad | Borazjan | Bushehr | 51.2605813 | 29.09663446 |
| 3 | Moraceae | 0 | Samal Abad | Borazjan | Bushehr | 51.2605813 | 29.09663446 |
| 4 | Arecaceae | 0 | Abad Natural Resources Forest | Borazjan | Bushehr | 51.2611708 | 29.06963713 |
| 4 | Asteraceae | 5 | Abad Natural Resources Forest | Borazjan | Bushehr | 51.2611708 | 29.06963713 |
| 4 | Cucurbitaceae | 0 | Abad Natural Resources Forest | Borazjan | Bushehr | 51.2611708 | 29.06963713 |
| 4 | Fabaceae | 64 | Abad Natural Resources Forest | Borazjan | Bushehr | 51.2611708 | 29.06963713 |
| 4 | Lamiaceae | 0 | Abad Natural Resources Forest | Borazjan | Bushehr | 51.2611708 | 29.06963713 |
| 4 | Poaceae | 128 | Abad Natural Resources Forest | Borazjan | Bushehr | 51.2611708 | 29.06963713 |
| 4 | Rhamnaceae | 0 | Abad Natural Resources Forest | Borazjan | Bushehr | 51.2611708 | 29.06963713 |
| 4 | Moraceae | 0 | Abad Natural Resources Forest | Borazjan | Bushehr | 51.2611708 | 29.06963713 |
| 5 | Arecaceae | 0 | Agha Shirmohammad-Kidi Valley | Borazjan | Bushehr | 51.2845721 | 29.06875015 |
| 5 | Asteraceae | 0 | Agha Shirmohammad-Kidi Valley | Borazjan | Bushehr | 51.2845721 | 29.06875015 |
| 5 | Cucurbitaceae | 0 | Agha Shirmohammad-Kidi Valley | Borazjan | Bushehr | 51.2845721 | 29.06875015 |
| 5 | Fabaceae | 0 | Agha Shirmohammad-Kidi Valley | Borazjan | Bushehr | 51.2845721 | 29.06875015 |
| 5 | Lamiaceae | 0 | Agha Shirmohammad-Kidi Valley | Borazjan | Bushehr | 51.2845721 | 29.06875015 |
| 5 | Poaceae | 0 | Agha Shirmohammad-Kidi Valley | Borazjan | Bushehr | 51.2845721 | 29.06875015 |
| 5 | Rhamnaceae | 0 | Agha Shirmohammad-Kidi Valley | Borazjan | Bushehr | 51.2845721 | 29.06875015 |
| 5 | Moraceae | 0 | Agha Shirmohammad-Kidi Valley | Borazjan | Bushehr | 51.2845721 | 29.06875015 |
| 6 | Arecaceae | 0 | Samal-Abanbar Road | Borazjan | Bushehr | 51.4375342 | 29.13143734 |
| 6 | Asteraceae | 1 | Samal-Abanbar Road | Borazjan | Bushehr | 51.4375342 | 29.13143734 |
| 6 | Cucurbitaceae | 0 | Samal-Abanbar Road | Borazjan | Bushehr | 51.4375342 | 29.13143734 |
| 6 | Fabaceae | 0 | Samal-Abanbar Road | Borazjan | Bushehr | 51.4375342 | 29.13143734 |
| 6 | Lamiaceae | 0 | Samal-Abanbar Road | Borazjan | Bushehr | 51.4375342 | 29.13143734 |
| 6 | Poaceae | 7 | Samal-Abanbar Road | Borazjan | Bushehr | 51.4375342 | 29.13143734 |
| 6 | Rhamnaceae | 7 | Samal-Abanbar Road | Borazjan | Bushehr | 51.4375342 | 29.13143734 |
| 6 | Moraceae | 0 | Samal-Abanbar Road | Borazjan | Bushehr | 51.4375342 | 29.13143734 |
| 7 | Arecaceae | 0 | Shirmard-Abparse Valley | Borazjan | Bushehr | 51.2841827 | 29.06911201 |
| 7 | Asteraceae | 6 | Shirmard-Abparse Valley | Borazjan | Bushehr | 51.2841827 | 29.06911201 |
| 7 | Cucurbitaceae | 0 | Shirmard-Abparse Valley | Borazjan | Bushehr | 51.2841827 | 29.06911201 |
| 7 | Fabaceae | 0 | Shirmard-Abparse Valley | Borazjan | Bushehr | 51.2841827 | 29.06911201 |
| 7 | Lamiaceae | 0 | Shirmard-Abparse Valley | Borazjan | Bushehr | 51.2841827 | 29.06911201 |
| 7 | Poaceae | 29 | Shirmard-Abparse Valley | Borazjan | Bushehr | 51.2841827 | 29.06911201 |
| 7 | Rhamnaceae | 0 | Shirmard-Abparse Valley | Borazjan | Bushehr | 51.2841827 | 29.06911201 |
| 7 | Moraceae | 0 | Shirmard-Abparse Valley | Borazjan | Bushehr | 51.2841827 | 29.06911201 |
| 8 | Arecaceae | 1 | Khate Luleh Padivar Road | Borazjan | Bushehr | 51.2660556 | 29.11942282 |
| 8 | Asteraceae | 0 | Khate Luleh Padivar Road | Borazjan | Bushehr | 51.2660556 | 29.11942282 |
| 8 | Cucurbitaceae | 0 | Khate Luleh Padivar Road | Borazjan | Bushehr | 51.2660556 | 29.11942282 |
| 8 | Fabaceae | 0 | Khate Luleh Padivar Road | Borazjan | Bushehr | 51.2660556 | 29.11942282 |
| 8 | Lamiaceae | 0 | Khate Luleh Padivar Road | Borazjan | Bushehr | 51.2660556 | 29.11942282 |
| 8 | Poaceae | 7 | Khate Luleh Padivar Road | Borazjan | Bushehr | 51.2660556 | 29.11942282 |
| 8 | Rhamnaceae | 33 | Khate Luleh Padivar Road | Borazjan | Bushehr | 51.2660556 | 29.11942282 |
| 8 | Moraceae | 0 | Khate Luleh Padivar Road | Borazjan | Bushehr | 51.2660556 | 29.11942282 |
| 9 | Arecaceae | 0 | Agha Shirmohammad Road | Borazjan | Bushehr | 51.2487599 | 29.06956142 |
| 9 | Asteraceae | 2 | Agha Shirmohammad Road | Borazjan | Bushehr | 51.2487599 | 29.06956142 |
| 9 | Cucurbitaceae | 0 | Agha Shirmohammad Road | Borazjan | Bushehr | 51.2487599 | 29.06956142 |
| 9 | Fabaceae | 0 | Agha Shirmohammad Road | Borazjan | Bushehr | 51.2487599 | 29.06956142 |
| 9 | Lamiaceae | 0 | Agha Shirmohammad Road | Borazjan | Bushehr | 51.2487599 | 29.06956142 |
| 9 | Poaceae | 22 | Agha Shirmohammad Road | Borazjan | Bushehr | 51.2487599 | 29.06956142 |
| 9 | Rhamnaceae | 0 | Agha Shirmohammad Road | Borazjan | Bushehr | 51.2487599 | 29.06956142 |
| 9 | Moraceae | 0 | Agha Shirmohammad Road | Borazjan | Bushehr | 51.2487599 | 29.06956142 |
| 10 | Arecaceae | 0 | Gas Station | Borazjan | Bushehr | 51.259586 | 29.10134785 |
| 10 | Asteraceae | 24 | Gas Station | Borazjan | Bushehr | 51.259586 | 29.10134785 |
| 10 | Cucurbitaceae | 0 | Gas Station | Borazjan | Bushehr | 51.259586 | 29.10134785 |
| 10 | Fabaceae | 1 | Gas Station | Borazjan | Bushehr | 51.259586 | 29.10134785 |
| 10 | Lamiaceae | 0 | Gas Station | Borazjan | Bushehr | 51.259586 | 29.10134785 |
| 10 | Poaceae | 3 | Gas Station | Borazjan | Bushehr | 51.259586 | 29.10134785 |
| 10 | Rhamnaceae | 0 | Gas Station | Borazjan | Bushehr | 51.259586 | 29.10134785 |
| 10 | Moraceae | 0 | Gas Station | Borazjan | Bushehr | 51.259586 | 29.10134785 |
| 11 | Arecaceae | 0 | Didashkan Valley | Borazjan | Bushehr | 51.2601952 | 29.09839524 |
| 11 | Asteraceae | 33 | Didashkan Valley | Borazjan | Bushehr | 51.2601952 | 29.09839524 |
| 11 | Cucurbitaceae | 0 | Didashkan Valley | Borazjan | Bushehr | 51.2601952 | 29.09839524 |
| 11 | Fabaceae | 93 | Didashkan Valley | Borazjan | Bushehr | 51.2601952 | 29.09839524 |
| 11 | Lamiaceae | 0 | Didashkan Valley | Borazjan | Bushehr | 51.2601952 | 29.09839524 |
| 11 | Poaceae | 1 | Didashkan Valley | Borazjan | Bushehr | 51.2601952 | 29.09839524 |
| 11 | Rhamnaceae | 0 | Didashkan Valley | Borazjan | Bushehr | 51.2601952 | 29.09839524 |
| 11 | Moraceae | 0 | Didashkan Valley | Borazjan | Bushehr | 51.2601952 | 29.09839524 |
| 12 | Arecaceae | 0 | Top Of Abad-Aghashirmorad Road | Borazjan | Bushehr | 51.2616874 | 29.0910995 |
| 12 | Asteraceae | 0 | Top Of Abad-Aghashirmorad Road | Borazjan | Bushehr | 51.2616874 | 29.0910995 |
| 12 | Cucurbitaceae | 4 | Top Of Abad-Aghashirmorad Road | Borazjan | Bushehr | 51.2616874 | 29.0910995 |
| 12 | Fabaceae | 0 | Top Of Abad-Aghashirmorad Road | Borazjan | Bushehr | 51.2616874 | 29.0910995 |
| 12 | Lamiaceae | 0 | Top Of Abad-Aghashirmorad Road | Borazjan | Bushehr | 51.2616874 | 29.0910995 |
| 12 | Poaceae | 0 | Top Of Abad-Aghashirmorad Road | Borazjan | Bushehr | 51.2616874 | 29.0910995 |
| 12 | Rhamnaceae | 9 | Top Of Abad-Aghashirmorad Road | Borazjan | Bushehr | 51.2616874 | 29.0910995 |
| 12 | Moraceae | 0 | Top Of Abad-Aghashirmorad Road | Borazjan | Bushehr | 51.2616874 | 29.0910995 |
| 13 | Arecaceae | 0 | Ghalat Road | Borazjan | Bushehr | 51.2740354 | 29.07419659 |
| 13 | Asteraceae | 71 | Ghalat Road | Borazjan | Bushehr | 51.2740354 | 29.07419659 |
| 13 | Cucurbitaceae | 0 | Ghalat Road | Borazjan | Bushehr | 51.2740354 | 29.07419659 |
| 13 | Fabaceae | 2 | Ghalat Road | Borazjan | Bushehr | 51.2740354 | 29.07419659 |
| 13 | Lamiaceae | 0 | Ghalat Road | Borazjan | Bushehr | 51.2740354 | 29.07419659 |
| 13 | Poaceae | 1 | Ghalat Road | Borazjan | Bushehr | 51.2740354 | 29.07419659 |
| 13 | Rhamnaceae | 0 | Ghalat Road | Borazjan | Bushehr | 51.2740354 | 29.07419659 |
| 13 | Moraceae | 0 | Ghalat Road | Borazjan | Bushehr | 51.2740354 | 29.07419659 |
| 14 | Arecaceae | 0 | Borazjan | Borazjan | Bushehr | 51.2482096 | 29.11857252 |
| 14 | Asteraceae | 6 | Borazjan | Borazjan | Bushehr | 51.2482096 | 29.11857252 |
| 14 | Cucurbitaceae | 0 | Borazjan | Borazjan | Bushehr | 51.2482096 | 29.11857252 |
| 14 | Fabaceae | 23 | Borazjan | Borazjan | Bushehr | 51.2482096 | 29.11857252 |
| 14 | Lamiaceae | 0 | Borazjan | Borazjan | Bushehr | 51.2482096 | 29.11857252 |
| 14 | Poaceae | 9 | Borazjan | Borazjan | Bushehr | 51.2482096 | 29.11857252 |
| 14 | Rhamnaceae | 0 | Borazjan | Borazjan | Bushehr | 51.2482096 | 29.11857252 |
| 14 | Moraceae | 33 | Borazjan | Borazjan | Bushehr | 51.2482096 | 29.11857252 |
| 15 | Arecaceae | 0 | Ghanat-Khate Luleh | Borazjan | Bushehr | 51.2627539 | 29.0861964 |
| 15 | Asteraceae | 2 | Ghanat-Khate Luleh | Borazjan | Bushehr | 51.2627539 | 29.0861964 |
| 15 | Cucurbitaceae | 0 | Ghanat-Khate Luleh | Borazjan | Bushehr | 51.2627539 | 29.0861964 |
| 15 | Fabaceae | 1 | Ghanat-Khate Luleh | Borazjan | Bushehr | 51.2627539 | 29.0861964 |
| 15 | Lamiaceae | 0 | Ghanat-Khate Luleh | Borazjan | Bushehr | 51.2627539 | 29.0861964 |
| 15 | Poaceae | 10 | Ghanat-Khate Luleh | Borazjan | Bushehr | 51.2627539 | 29.0861964 |
| 15 | Rhamnaceae | 3 | Ghanat-Khate Luleh | Borazjan | Bushehr | 51.2627539 | 29.0861964 |
| 15 | Moraceae | 0 | Ghanat-Khate Luleh | Borazjan | Bushehr | 51.2627539 | 29.0861964 |
| 16 | Arecaceae | 0 | Natural Resources Forest | Borazjan | Bushehr | 51.2319548 | 29.07079195 |
| 16 | Asteraceae | 0 | Natural Resources Forest | Borazjan | Bushehr | 51.2319548 | 29.07079195 |
| 16 | Cucurbitaceae | 0 | Natural Resources Forest | Borazjan | Bushehr | 51.2319548 | 29.07079195 |
| 16 | Fabaceae | 1 | Natural Resources Forest | Borazjan | Bushehr | 51.2319548 | 29.07079195 |
| 16 | Lamiaceae | 0 | Natural Resources Forest | Borazjan | Bushehr | 51.2319548 | 29.07079195 |
| 16 | Poaceae | 2 | Natural Resources Forest | Borazjan | Bushehr | 51.2319548 | 29.07079195 |
| 16 | Rhamnaceae | 0 | Natural Resources Forest | Borazjan | Bushehr | 51.2319548 | 29.07079195 |
| 16 | Moraceae | 0 | Natural Resources Forest | Borazjan | Bushehr | 51.2319548 | 29.07079195 |
| 17 | Arecaceae | 0 | Top of Abad-Khate Luleh Road | Borazjan | Bushehr | 51.2314075 | 29.10632751 |
| 17 | Asteraceae | 4 | Top of Abad-Khate Luleh Road | Borazjan | Bushehr | 51.2314075 | 29.10632751 |
| 17 | Cucurbitaceae | 0 | Top of Abad-Khate Luleh Road | Borazjan | Bushehr | 51.2314075 | 29.10632751 |
| 17 | Fabaceae | 0 | Top of Abad-Khate Luleh Road | Borazjan | Bushehr | 51.2314075 | 29.10632751 |
| 17 | Lamiaceae | 0 | Top of Abad-Khate Luleh Road | Borazjan | Bushehr | 51.2314075 | 29.10632751 |
| 17 | Poaceae | 5 | Top of Abad-Khate Luleh Road | Borazjan | Bushehr | 51.2314075 | 29.10632751 |
| 17 | Rhamnaceae | 0 | Top of Abad-Khate Luleh Road | Borazjan | Bushehr | 51.2314075 | 29.10632751 |
| 17 | Moraceae | 0 | Top of Abad-Khate Luleh Road | Borazjan | Bushehr | 51.2314075 | 29.10632751 |
| 18 | Arecaceae | 0 | Samal-Khate Luleh Road | Borazjan | Bushehr | 51.2587737 | 29.10531174 |
| 18 | Asteraceae | 0 | Samal-Khate Luleh Road | Borazjan | Bushehr | 51.2587737 | 29.10531174 |
| 18 | Cucurbitaceae | 0 | Samal-Khate Luleh Road | Borazjan | Bushehr | 51.2587737 | 29.10531174 |
| 18 | Fabaceae | 0 | Samal-Khate Luleh Road | Borazjan | Bushehr | 51.2587737 | 29.10531174 |
| 18 | Lamiaceae | 0 | Samal-Khate Luleh Road | Borazjan | Bushehr | 51.2587737 | 29.10531174 |
| 18 | Poaceae | 40 | Samal-Khate Luleh Road | Borazjan | Bushehr | 51.2587737 | 29.10531174 |
| 18 | Rhamnaceae | 0 | Samal-Khate Luleh Road | Borazjan | Bushehr | 51.2587737 | 29.10531174 |
| 18 | Moraceae | 0 | Samal-Khate Luleh Road | Borazjan | Bushehr | 51.2587737 | 29.10531174 |
| 19 | Arecaceae | 0 | Mohammad Salehi | Borazjan | Bushehr | 50.61412 | 29.65822 |
| 19 | Asteraceae | 0 | Mohammad Salehi | Borazjan | Bushehr | 50.61412 | 29.65822 |
| 19 | Cucurbitaceae | 0 | Mohammad Salehi | Borazjan | Bushehr | 50.61412 | 29.65822 |
| 19 | Fabaceae | 0 | Mohammad Salehi | Borazjan | Bushehr | 50.61412 | 29.65822 |
| 19 | Lamiaceae | 0 | Mohammad Salehi | Borazjan | Bushehr | 50.61412 | 29.65822 |
| 19 | Poaceae | 6 | Mohammad Salehi | Borazjan | Bushehr | 50.61412 | 29.65822 |
| 19 | Rhamnaceae | 0 | Mohammad Salehi | Borazjan | Bushehr | 50.61412 | 29.65822 |
| 19 | Moraceae | 0 | Mohammad Salehi | Borazjan | Bushehr | 50.61412 | 29.65822 |
| 20 | Arecaceae | 0 | Jade Miani, Nokan Village | Borazjan | Bushehr | 51.77072 | 27.97998 |
| 20 | Asteraceae | 0 | Jade Miani, Nokan Village | Borazjan | Bushehr | 51.77072 | 27.97998 |
| 20 | Cucurbitaceae | 0 | Jade Miani, Nokan Village | Borazjan | Bushehr | 51.77072 | 27.97998 |
| 20 | Fabaceae | 0 | Jade Miani, Nokan Village | Borazjan | Bushehr | 51.77072 | 27.97998 |
| 20 | Lamiaceae | 0 | Jade Miani, Nokan Village | Borazjan | Bushehr | 51.77072 | 27.97998 |
| 20 | Poaceae | 0 | Jade Miani, Nokan Village | Borazjan | Bushehr | 51.77072 | 27.97998 |
| 20 | Rhamnaceae | 0 | Jade Miani, Nokan Village | Borazjan | Bushehr | 51.77072 | 27.97998 |
| 20 | Moraceae | 0 | Jade Miani, Nokan Village | Borazjan | Bushehr | 51.77072 | 27.97998 |
| 21 | Arecaceae | 0 | Meshrage | Borazjan | Bushehr | 49.79487 | 30.54701 |
| 21 | Asteraceae | 19 | Meshrage | Borazjan | Bushehr | 49.79487 | 30.54701 |
| 21 | Cucurbitaceae | 0 | Meshrage | Borazjan | Bushehr | 49.79487 | 30.54701 |
| 21 | Fabaceae | 4 | Meshrage | Borazjan | Bushehr | 49.79487 | 30.54701 |
| 21 | Lamiaceae | 0 | Meshrage | Borazjan | Bushehr | 49.79487 | 30.54701 |
| 21 | Poaceae | 226 | Meshrage | Borazjan | Bushehr | 49.79487 | 30.54701 |
| 21 | Rhamnaceae | 0 | Meshrage | Borazjan | Bushehr | 49.79487 | 30.54701 |
| 21 | Moraceae | 0 | Meshrage | Borazjan | Bushehr | 49.79487 | 30.54701 |
| 22 | Arecaceae | 0 | Dopiran-Gotvand | Borazjan | Bushehr | 48.86951 | 32.11855 |
| 22 | Asteraceae | 0 | Dopiran-Gotvand | Borazjan | Bushehr | 48.86951 | 32.11855 |
| 22 | Cucurbitaceae | 0 | Dopiran-Gotvand | Borazjan | Bushehr | 48.86951 | 32.11855 |
| 22 | Fabaceae | 36 | Dopiran-Gotvand | Borazjan | Bushehr | 48.86951 | 32.11855 |
| 22 | Lamiaceae | 0 | Dopiran-Gotvand | Borazjan | Bushehr | 48.86951 | 32.11855 |
| 22 | Poaceae | 0 | Dopiran-Gotvand | Borazjan | Bushehr | 48.86951 | 32.11855 |
| 22 | Rhamnaceae | 0 | Dopiran-Gotvand | Borazjan | Bushehr | 48.86951 | 32.11855 |
| 22 | Moraceae | 0 | Dopiran-Gotvand | Borazjan | Bushehr | 48.86951 | 32.11855 |
| 23 | Arecaceae | 0 | Paeen Tape Village | Borazjan | Bushehr | 47.77884 | 32.67749 |
| 23 | Asteraceae | 0 | Paeen Tape Village | Borazjan | Bushehr | 47.77884 | 32.67749 |
| 23 | Cucurbitaceae | 0 | Paeen Tape Village | Borazjan | Bushehr | 47.77884 | 32.67749 |
| 23 | Fabaceae | 0 | Paeen Tape Village | Borazjan | Bushehr | 47.77884 | 32.67749 |
| 23 | Lamiaceae | 0 | Paeen Tape Village | Borazjan | Bushehr | 47.77884 | 32.67749 |
| 23 | Poaceae | 0 | Paeen Tape Village | Borazjan | Bushehr | 47.77884 | 32.67749 |
| 23 | Rhamnaceae | 0 | Paeen Tape Village | Borazjan | Bushehr | 47.77884 | 32.67749 |
| 23 | Moraceae | 0 | Paeen Tape Village | Borazjan | Bushehr | 47.77884 | 32.67749 |
| 24 | Arecaceae | 0 | Mormori | Borazjan | Bushehr | 47.78064 | 32.67858 |
| 24 | Asteraceae | 9 | Mormori | Borazjan | Bushehr | 47.78064 | 32.67858 |
| 24 | Cucurbitaceae | 0 | Mormori | Borazjan | Bushehr | 47.78064 | 32.67858 |
| 24 | Fabaceae | 59 | Mormori | Borazjan | Bushehr | 47.78064 | 32.67858 |
| 24 | Lamiaceae | 0 | Mormori | Borazjan | Bushehr | 47.78064 | 32.67858 |
| 24 | Poaceae | 29 | Mormori | Borazjan | Bushehr | 47.78064 | 32.67858 |
| 24 | Rhamnaceae | 0 | Mormori | Borazjan | Bushehr | 47.78064 | 32.67858 |
| 24 | Moraceae | 0 | Mormori | Borazjan | Bushehr | 47.78064 | 32.67858 |

Table S3. Number of seeds for each plant family in each fecal pellet group during May to July 2016.

| No. Pellet | Lizard Number | Food Items | Plant Family | Number of Seeds |
| --- | --- | --- | --- | --- |
| 1 | 1 | Gastropoda |  |  |
| 1 | 1 | Small mammal |  |  |
| 1 | 1 | Date palm fruit | Arecaceae | 1 |
| 1 | 1 | Poaceae | Poaceae | 0 |
| 1 | 1 | Insecta |  |  |
| 1 | 1 | Coleoptera |  |  |
| 2 | 1 | Coleoptera |  |  |
| 2 | 1 | *Medicago polymorpha* | Fabaceae | 1 |
| 2 | 1 | *Centaurea* sp. | Asteraceae | 1 |
| 3 | 1 | *Centaurea* sp. | Asteraceae | 0 |
| 3 | 1 | Poaceae | Poaceae | 0 |
| 4 | 1 | Small mammal |  |  |
| 4 | 1 | *Ziziphus spina-christi* | Rhamnaceae | 3 |
| 4 | 1 | Poaceae | Poaceae | 0 |
| 4 | 1 | *Centaurea* sp. | Asteraceae | 0 |
| 5 | 2 | *Centaurea* sp. | Asteraceae | 8 |
| 5 | 2 | Coleoptera |  |  |
| 6 | 2 | Coleoptera |  |  |
| 6 | 2 | *Centaurea* sp. | Asteraceae | 1 |
| 6 | 2 | Poaceae | Poaceae | 7 |
| 7 | 2 | *Centaurea* sp. | Asteraceae | 0 |
| 7 | 2 | Poaceae | Poaceae | 2 |
| 8 | 2 | Small mammal |  |  |
| 8 | 2 | *Centaurea* sp. | Asteraceae | 16 |
| 8 | 2 | Coleoptera |  |  |
| 8 | 2 | Poaceae | Poaceae | 9 |
| 9 | 3 | *Centaurea* sp. | Asteraceae | 10 |
| 9 | 3 | Poaceae | Poaceae | 4 |
| 10 | 4 | Formicidae |  |  |
| 10 | 4 | *Centaurea* sp. | Asteraceae | 0 |
| 10 | 4 | Fabaceae | Fabaceae | 11 |
| 10 | 4 | Coleoptera |  |  |
| 11 | 4 | *Centaurea* sp. | Asteraceae | 3 |
| 11 | 4 | Coleoptera |  |  |
| 11 | 4 | Fabaceae | Fabaceae | 14 |
| 12 | 4 | Coleoptera |  |  |
| 12 | 4 | *Centaurea* sp. | Asteraceae | 0 |
| 12 | 4 | Fabaceae | Fabaceae | 23 |
| 13 | 4 | Coleoptera |  |  |
| 13 | 4 | Poaceae | Poaceae | 7 |
| 14 | 4 | Coleoptera |  |  |
| 14 | 4 | Fabaceae | Fabaceae | 9 |
| 14 | 4 | Lamiaceae | Lamiaceae | 0 |
| 14 | 4 | Dandelion, *Taraxacum* sp. | Asteraceae | 0 |
| 15 | 4 | Dandelion, *Taraxacum* sp. | Asteraceae | 0 |
| 15 | 4 | Coleoptera |  |  |
| 16 | 4 | Coleoptera |  |  |
| 16 | 4 | Poaceae | Poaceae | 121 |
| 17 | 4 | *Centaurea* sp. | Asteraceae | 2 |
| 17 | 4 | *Prosopis juliflora* | Fabaceae | 1 |
| 17 | 4 | Fabaceae | Fabaceae | 1 |
| 18 | 4 | Coleoptera |  |  |
| 18 | 4 | Fabaceae | Fabaceae | 3 |
| 19 | 4 | Coleoptera |  |  |
| 19 | 4 | Dandelion, *Taraxacum* sp. | Asteraceae | 0 |
| 19 | 4 | Fabaceae | Fabaceae | 2 |
| 19 | 4 | Poaceae | Poaceae | 0 |
| 19 | 4 | Poaceae | Poaceae | 0 |
| 20 | 5 | Poaceae | Poaceae | 0 |
| 20 | 5 | *Centaurea* sp. | Asteraceae | 0 |
| 22 | 5 | Formicidae |  |  |
| 22 | 5 | Poaceae | Poaceae | 0 |
| 23 | 6 | Poaceae | Poaceae | 5 |
| 23 | 6 | Coleoptera |  |  |
| 24 | 6 | Rhamnaceae Fruits | Rhamnaceae | 3 |
| 24 | 6 | Ground beetles, Carabidae |  |  |
| 24 | 6 | Coleoptera |  |  |
| 24 | 6 | Poaceae | Poaceae | 2 |
| 25 | 6 | Ground beetles, Carabidae |  |  |
| 25 | 6 | Small scorpion, Scorpiones |  |  |
| 25 | 6 | *Centaurea* sp. | Asteraceae | 1 |
| 25 | 6 | Rhamnaceae Fruits | Rhamnaceae | 1 |
| 25 | 6 | True weevil, Curculionidae |  |  |
| 21 | 6 | Rhamnaceae Fruits | Rhamnaceae | 3 |
| 21 | 6 | Coleoptera |  |  |
| 26 | 7 | Asteraceae | Asteraceae | 6 |
| 27 | 7 | Poaceae | Poaceae | 6 |
| 28 | 7 | Asteraceae | Asteraceae | 0 |
| 28 | 7 | *Centaurea* sp. | Asteraceae | 0 |
| 28 | 7 | Coleoptera |  |  |
| 29 | 7 | Poaceae | Poaceae | 23 |
| 30 | 8 | Date palm fruit | Arecaceae | 1 |
| 30 | 8 | Poaceae | Poaceae | 0 |
| 30 | 8 | *Ziziphus spina-christi* | Rhamnaceae | 1 |
| 30 | 8 | Coleoptera |  |  |
| 30 | 8 | Cockroach, Dictyoptera |  |  |
| 31 | 8 | *Ziziphus spina-christi* | Rhamnaceae | 13 |
| 31 | 8 | Coleoptera |  |  |
| 31 | 8 | Insecta |  |  |
| 31 | 8 | Poaceae | Poaceae | 1 |
| 32 | 8 | Small mammal |  |  |
| 32 | 8 | Coleoptera |  |  |
| 32 | 8 | Poaceae | Poaceae | 2 |
| 32 | 8 | Insecta |  |  |
| 32 | 8 | *Ziziphus spina-christi* | Rhamnaceae | 19 |
| 32 | 8 | Formicidae |  |  |
| 32 | 8 | Poaceae | Poaceae | 4 |
| 33 | 9 | *Centaurea* sp. | Asteraceae | 2 |
| 33 | 9 | Poaceae | Poaceae | 17 |
| 34 | 9 | *Centaurea* sp. | Asteraceae | 0 |
| 35 | 9 | Poaceae | Poaceae | 5 |
| 36 | 10 | Asteraceae | Asteraceae | 0 |
| 36 | 10 | *Medicago polymorpha* | Fabaceae | 1 |
| 36 | 10 | *Centaurea* sp. | Asteraceae | 3 |
| 37 | 10 | *Centaurea* sp. | Asteraceae | 12 |
| 37 | 10 | Poaceae | Poaceae | 3 |
| 38 | 10 | *Centaurea* sp. | Asteraceae | 9 |
| 39 | 10 | *Centaurea* sp. | Asteraceae | 0 |
| 40 | 10 | *Centaurea* sp. | Asteraceae | 0 |
| 41 | 10 | *Centaurea* sp. | Asteraceae | 0 |
| 42 | 10 | *Centaurea* sp. | Asteraceae | 0 |
| 43 | 11 | *Centaurea* sp. | Asteraceae | 0 |
| 43 | 11 | Coleoptera |  |  |
| 43 | 11 | Fabaceae | Fabaceae | 17 |
| 44 | 11 | *Centaurea* sp. | Asteraceae | 0 |
| 44 | 11 | *Medicago polymorpha* | Fabaceae | 3 |
| 44 | 11 | Fabaceae | Fabaceae | 24 |
| 44 | 11 | Coleoptera |  |  |
| 45 | 11 | *Centaurea* sp. | Asteraceae | 2 |
| 45 | 11 | *Medicago polymorpha* | Fabaceae | 6 |
| 45 | 11 | Coleoptera |  |  |
| 45 | 11 | Fabaceae | Fabaceae | 17 |
| 46 | 11 | Asteraceae | Asteraceae | 24 |
| 46 | 11 | *Centaurea* sp. | Asteraceae | 4 |
| 46 | 11 | *Medicago polymorpha* | Fabaceae | 12 |
| 47 | 11 | Asteraceae | Asteraceae | 1 |
| 47 | 11 | *Centaurea* sp. | Asteraceae | 1 |
| 47 | 11 | Fabaceae | Fabaceae | 4 |
| 47 | 11 | *Medicago polymorpha* | Fabaceae | 2 |
| 47 | 11 | Poaceae | Poaceae | 1 |
| 48 | 11 | *Medicago polymorpha* | Fabaceae | 3 |
| 48 | 11 | Fabaceae | Fabaceae | 5 |
| 48 | 11 | *Centaurea* sp. | Asteraceae | 1 |
| 49 | 12 | *Ziziphus spina-christi* | Rhamnaceae | 9 |
| 49 | 12 | Coleoptera |  |  |
| 49 | 12 | Muskmelons, *Cucumis melo* | Cucurbitaceae | 4 |
| 50 | 13 | *Centaurea* sp. | Asteraceae | 1 |
| 50 | 13 | Poaceae | Poaceae | 0 |
| 51 | 13 | *Centaurea* sp. | Asteraceae | 34 |
| 51 | 13 | Poaceae | Poaceae | 1 |
| 52 | 13 | *Centaurea* sp. | Asteraceae | 0 |
| 53 | 13 | Asteraceae | Asteraceae | 7 |
| 53 | 13 | *Centaurea* sp. | Asteraceae | 16 |
| 53 | 13 | Fabaceae | Fabaceae | 1 |
| 53 | 13 | *Medicago polymorpha* | Fabaceae | 1 |
| 54 | 13 | *Centaurea* sp. | Asteraceae | 13 |
| 54 | 13 | Poaceae | Poaceae | 0 |
| 55 | 14 | *Centaurea* sp. | Asteraceae | 2 |
| 55 | 14 | *Medicago polymorpha* | Fabaceae | 7 |
| 55 | 14 | Poaceae | Poaceae | 1 |
| 55 | 14 | Fig fruits, *Ficus carica* | Moraceae | 33 |
| 56 | 14 | Poaceae | Poaceae | 4 |
| 56 | 14 | *Centaurea* sp. | Asteraceae | 2 |
| 57 | 14 | *Medicago polymorpha* | Fabaceae | 7 |
| 57 | 14 | Poaceae | Poaceae | 4 |
| 58 | 14 | *Medicago polymorpha* | Fabaceae | 1 |
| 59 | 14 | Ground beetles, Carabidae |  |  |
| 59 | 14 | *Medicago polymorpha* | Fabaceae | 1 |
| 59 | 14 | Poaceae | Poaceae | 0 |
| 59 | 14 | *Centaurea* sp. | Asteraceae | 1 |
| 60 | 14 | *Medicago polymorpha* | Fabaceae | 3 |
| 61 | 14 | Fabaceae | Fabaceae | 1 |
| 61 | 14 | *Medicago polymorpha* | Fabaceae | 2 |
| 62 | 14 | *Medicago polymorpha* | Fabaceae | 1 |
| 63 | 14 | *Centaurea* sp. | Asteraceae | 1 |
| 64 | 15 | Coleoptera |  |  |
| 64 | 15 | Poaceae | Poaceae | 7 |
| 64 | 15 | *Medicago polymorpha* | Fabaceae | 1 |
| 64 | 15 | *Centaurea* sp. | Asteraceae | 1 |
| 64 | 15 | *Ziziphus spina-christi* | Rhamnaceae | 2 |
| 65 | 15 | Coleoptera |  |  |
| 65 | 15 | *Centaurea* sp. | Asteraceae | 1 |
| 65 | 15 | Poaceae | Poaceae | 3 |
| 66 | 15 | *Ziziphus spina-christi* | Rhamnaceae | 1 |
| 66 | 15 | *Centaurea* sp. | Asteraceae | 0 |
| 67 | 16 | *Medicago polymorpha* | Fabaceae | 0 |
| 67 | 16 | *Centaurea* sp. | Asteraceae | 0 |
| 67 | 16 | Poaceae | Poaceae | 2 |
| 68 | 16 | *Medicago polymorpha* | Fabaceae | 1 |
| 68 | 16 | Coleoptera |  |  |
| 68 | 16 | *Centaurea* sp. | Asteraceae | 0 |
| 69 | 17 | *Centaurea* sp. | Asteraceae | 4 |
| 69 | 17 | Poaceae | Poaceae | 5 |
| 70 | 18 | Poaceae | Poaceae | 12 |
| 71 | 18 | Poaceae | Poaceae | 15 |
| 72 | 18 | Poaceae | Poaceae | 1 |
| 73 | 18 | Poaceae | Poaceae | 12 |
| 74 | 18 | Poaceae | Poaceae | 0 |
| 75 | 19 | Jewel beetle, *Julodis* sp. |  |  |
| 75 | 19 | Poaceae | Poaceae | 6 |
| 76 | 20 | Coleoptera |  |  |
| 76 | 20 | Poaceae | Poaceae | 0 |
| 77 | 21 | Coleoptera |  |  |
| 77 | 21 | *Medicago polymorpha* | Fabaceae | 0 |
| 77 | 21 | *Bromus* sp. | Poaceae | 5 |
| 77 | 21 | Poaceae | Poaceae | 0 |
| 78 | 21 | *Bromus* sp. | Poaceae | 7 |
| 79 | 21 | Asteraceae | Asteraceae | 0 |
| 79 | 21 | *Medicago polymorpha* | Fabaceae | 1 |
| 79 | 21 | *Bromus* sp. | Poaceae | 9 |
| 79 | 21 | Ground beetles, Carabidae |  |  |
| 79 | 21 | Coleoptera |  |  |
| 79 | 21 | Poaceae | Poaceae | 0 |
| 80 | 21 | *Medicago polymorpha* | Fabaceae | 1 |
| 80 | 21 | *Bromus* sp. | Poaceae | 12 |
| 81 | 21 | Jewel beetle, *Julodis* sp. |  |  |
| 81 | 21 | Adesmia Beetle, *Adesmia* sp. |  |  |
| 81 | 21 | *Centaurea* sp. | Asteraceae | 0 |
| 82 | 21 | Coleoptera |  |  |
| 82 | 21 | Fly, Diptera |  |  |
| 83 | 21 | Coleoptera |  |  |
| 83 | 21 | *Bromus* sp. | Poaceae | 8 |
| 83 | 21 | Asteraceae | Asteraceae | 7 |
| 84 | 21 | Coleoptera |  |  |
| 84 | 21 | *Bromus* sp. | Poaceae | 11 |
| 84 | 21 | Asteraceae | Asteraceae | 7 |
| 84 | 21 | *Centaurea* sp. | Asteraceae | 0 |
| 85 | 21 | Asteraceae | Asteraceae | 3 |
| 86 | 21 | *Bromus* sp. | Poaceae | 39 |
| 86 | 21 | *Medicago polymorpha* | Fabaceae | 2 |
| 87 | 21 | *Bromus* sp. | Poaceae | 127 |
| 88 | 21 | *Bromus* sp. | Poaceae | 8 |
| 88 | 21 | Poaceae | Poaceae | 0 |
| 89 | 21 | *Centaurea* sp. | Asteraceae | 2 |
| 89 | 21 | *Medicago polymorpha* | Fabaceae | 0 |
| 90 | 21 | Formicidae |  |  |
| 90 | 21 | Poaceae | Poaceae | 0 |
| 91 | 21 | Asteraceae | Asteraceae | 0 |
| 92 | 22 | *Medicago polymorpha* | Fabaceae | 36 |
| 92 | 22 | *Bromus* sp. | Poaceae | 0 |
| 92 | 22 | *Centaurea* sp. | Asteraceae | 0 |
| 93 | 23 | Asteraceae | Asteraceae | 0 |
| 93 | 23 | Coleoptera |  |  |
| 94 | 24 | Common Wheat, *Triticum aestivum* | Poaceae | 3 |
| 95 | 24 | Poaceae | Poaceae | 1 |
| 96 | 24 | Poaceae | Poaceae | 1 |
| 97 | 24 | *Medicago polymorpha* | Fabaceae | 2 |
| 97 | 24 | Poaceae | Poaceae | 2 |
| 98 | 24 | *Medicago polymorpha* | Fabaceae | 3 |
| 98 | 24 | Common Wheat, *Triticum aestivum* | Poaceae | 1 |
| 99 | 24 | Barley, *Hordeum vulgare* | Poaceae | 2 |
| 99 | 24 | *Medicago polymorpha* | Fabaceae | 6 |
| 100 | 24 | Asteraceae | Asteraceae | 4 |
| 100 | 24 | Poaceae | Poaceae | 1 |
| 100 | 24 | *Medicago polymorpha* | Fabaceae | 1 |
| 101 | 24 | Poaceae | Poaceae | 5 |
| 101 | 24 | Coleoptera |  |  |
| 102 | 24 | *Centaurea* sp. | Asteraceae | 1 |
| 102 | 24 | Coleoptera |  |  |
| 102 | 24 | Poaceae | Poaceae | 1 |
| 103 | 24 | *Centaurea* sp. | Asteraceae | 1 |
| 104 | 24 | Poaceae | Poaceae | 0 |
| 105 | 24 | Poaceae | Poaceae | 1 |
| 105 | 24 | *Medicago polymorpha* | Fabaceae | 3 |
| 105 | 24 | Asteraceae | Asteraceae | 0 |
| 105 | 24 | Small mammal |  |  |
| 106 | 24 | Poaceae | Poaceae | 0 |
| 106 | 24 | Coleoptera |  |  |
| 107 | 24 | Poaceae | Poaceae | 0 |
| 107 | 24 | Asteraceae | Asteraceae | 1 |
| 107 | 24 | Coleoptera |  |  |
| 107 | 24 | Wasp, Vespidae |  |  |
| 108 | 24 | *Medicago polymorpha* | Fabaceae | 3 |
| 108 | 24 | Coleoptera |  |  |
| 108 | 24 | Poaceae | Poaceae | 0 |
| 109 | 24 | Fabaceae | Fabaceae | 1 |
| 110 | 24 | Poaceae | Poaceae | 0 |
| 111 | 24 | Coleoptera |  |  |
| 111 | 24 | Poaceae | Poaceae | 1 |
| 112 | 24 | *Medicago polymorpha* | Fabaceae | 3 |
| 112 | 24 | Poaceae | Poaceae | 0 |
| 112 | 24 | Coleoptera |  |  |
| 113 | 24 | Poaceae | Poaceae | 0 |
| 114 | 24 | Poaceae | Poaceae | 2 |
| 114 | 24 | Coleoptera |  |  |
| 115 | 24 | Poaceae | Poaceae | 2 |
| 115 | 24 | Coleoptera |  |  |
| 116 | 24 | Poaceae | Poaceae | 2 |
| 116 | 24 | Coleoptera |  |  |
| 117 | 24 | *Medicago polymorpha* | Fabaceae | 2 |
| 118 | 24 | Poaceae | Poaceae | 0 |
| 119 | 24 | Poaceae | Poaceae | 2 |
| 119 | 24 | Coleoptera |  |  |
| 119 | 24 | Small mammal |  |  |
| 119 | 24 | *Medicago polymorpha* | Fabaceae | 9 |
| 120 | 24 | Poaceae | Poaceae | 0 |
| 120 | 24 | Poaceae | Poaceae | 1 |
| 121 | 24 | *Medicago polymorpha* | Fabaceae | 26 |
| 122 | 24 | Coleoptera |  |  |
| 122 | 24 | *Centaurea* sp. | Asteraceae | 1 |
| 123 | 24 | *Centaurea* sp. | Asteraceae | 1 |
| 124 | 24 | Poaceae | Poaceae | 1 |
| 124 | 24 | *Centaurea* sp. | Asteraceae | 0 |

**Data Analysis 1**

data: FoodGroupV and Province

Kruskal-Wallis chi-squared = 4.0517, df = 2, p-value = 0.13

Comparison of FoodGroupV by Province

(Benjamini-Hochberg)

Col Mean-|

Row Mean | Bushehr Ilam

---------+----------------------

Ilam | -1.978166

| 0.0719

|

Khuzesta | -0.789787 0.744120

| 0.3222 0.2284

List of pairwise comparisons: Z statistic (adjusted p-value)

----------------------------------------

Bushehr - Ilam : -1.978166 (0.0719)

Bushehr - Khuzestan : -0.789787 (0.3222)

Ilam - Khuzestan : 0.744120 (0.2284)

alpha = 0.05

Reject Ho if p <= alpha/2

> ## Air quality data set illustrates differences in different

> ## multiple comparisons adjustments

> dt <- dunn.test(FoodGroupV, FoodGroup, kw=F, method="bonferroni")

Comparison of FoodGroupV by FoodGroup

(Bonferroni)

Col Mean-|

Row Mean | Crops Cultivat Herbs Inverteb Mammals

---------+-------------------------------------------------------

Cultivat | -0.073439

| 1.0000

|

Herbs | -0.197080 -0.074738

| 1.0000 1.0000

|

Inverteb | 2.462132 2.242491 9.606646

| 0.1036 0.1870 0.0000*

|

Mammals | 2.022707 1.925793 3.388461 0.090041

| 0.3233 0.4060 0.0053* 1.0000

|

Wild fru | 0.142642 0.213982 0.589504 -3.639459 -2.408512

| 1.0000 1.0000 1.0000 0.0020* 0.1201

alpha = 0.05

Reject Ho if p <= alpha/2

> ## Air quality data set illustrates differences in different

> ## multiple comparisons adjustments

> dt <- dunn.test(FoodGroupV, FoodGroup, kw=F, method="bonferroni")

**Data Analysis 2**

#------------Nonparametric Multiple Comparisons for relative contrast effects----------#

- Alternative Hypothesis: True relative contrast effect p is less than 1/2

- Estimation Method: Global Pseudo ranks

- Type of Contrast : Tukey

- Confidence Level: 95 %

- Method = Multi - T with 123 DF

- Estimation Method: Pairwise rankings

#---------------------------Interpretation--------------------------------------------#

p(a,b) > 1/2 : b tends to be larger than a

#-------------------------------------------------------------------------------------#

#----Data Info-------------------------------------------------------------------------#

Sample Size

1 Arecaceae 124

2 Asteraceae 124

3 Cucurbitaceae 124

4 Fabaceae 124

5 Lamiaceae 124

6 Moraceae 124

7 Poaceae 124

8 Rhamnaceae 124

#----Analysis--------------------------------------------------------------------------#

Comparison Estimator Lower Upper Statistic p.Value

1 p( Arecaceae , Asteraceae ) 0.647 0 0.707 6.827239021 1.000000e+00

2 p( Arecaceae , Cucurbitaceae ) 0.496 0 0.515 -0.569521686 0.000000e+00

3 p( Arecaceae , Fabaceae ) 0.655 0 0.716 7.127796608 1.000000e+00

4 p( Arecaceae , Lamiaceae ) 0.492 0 0.508 -1.419997691 0.000000e+00

5 p( Arecaceae , Moraceae ) 0.496 0 0.515 -0.569521686 0.000000e+00

6 p( Arecaceae , Poaceae ) 0.716 0 0.780 9.414032842 1.000000e+00

7 p( Arecaceae , Rhamnaceae ) 0.533 0 0.570 2.419295091 8.784472e-01

8 p( Asteraceae , Cucurbitaceae ) 0.351 0 0.410 -7.019454257 0.000000e+00

9 p( Asteraceae , Fabaceae ) 0.514 0 0.598 0.467251214 7.738163e-03

10 p( Asteraceae , Lamiaceae ) 0.347 0 0.405 -7.372166385 0.000000e+00

11 p( Asteraceae , Moraceae ) 0.352 0 0.411 -6.958650437 0.000000e+00

12 p( Asteraceae , Poaceae ) 0.578 0 0.666 2.469284266 8.917019e-01

13 p( Asteraceae , Rhamnaceae ) 0.388 0 0.456 -4.606164577 0.000000e+00

14 p( Cucurbitaceae , Fabaceae ) 0.657 0 0.717 7.310653431 1.000000e+00

15 p( Cucurbitaceae , Lamiaceae ) 0.496 0 0.507 -1.000000000 0.000000e+00

16 p( Cucurbitaceae , Moraceae ) 0.500 0 0.516 0.005702289 3.359984e-06

17 p( Cucurbitaceae , Poaceae ) 0.718 0 0.781 9.567332313 1.000000e+00

18 p( Cucurbitaceae , Rhamnaceae ) 0.536 0 0.572 2.797682992 9.521340e-01

19 p( Fabaceae , Lamiaceae ) 0.339 0 0.397 -7.653197278 0.000000e+00

20 p( Fabaceae , Moraceae ) 0.344 0 0.404 -7.234375283 0.000000e+00

21 p( Fabaceae , Poaceae ) 0.561 0 0.650 1.914545997 6.684581e-01

22 p( Fabaceae , Rhamnaceae ) 0.378 0 0.447 -4.964887801 0.000000e+00

23 p( Lamiaceae , Moraceae ) 0.504 0 0.515 1.000000000 1.218490e-01

24 p( Lamiaceae , Poaceae ) 0.722 0 0.784 9.901690677 1.000000e+00

25 p( Lamiaceae , Rhamnaceae ) 0.540 0 0.574 3.284733427 9.886312e-01

26 p( Moraceae , Poaceae ) 0.716 0 0.780 9.423357183 1.000000e+00

27 p( Moraceae , Rhamnaceae ) 0.536 0 0.572 2.780896545 9.502585e-01

28 p( Poaceae , Rhamnaceae ) 0.317 0 0.389 -7.097415829 0.000000e+00

#----Overall---------------------------------------------------------------------------#

Quantile p.Value

1 2.782783 0

#--------------------------------------------------------------------------------------#
